# Ferulic Acid: A Review of Pharmacology, Toxicology, and Therapeutic Effects on Pulmonary Diseases

**DOI:** 10.3390/ijms24098011

**Published:** 2023-04-28

**Authors:** Yiman Zhai, Tingyu Wang, Yunmei Fu, Tong Yu, Yan Ding, Hongguang Nie

**Affiliations:** Department of Stem Cells and Regenerative Medicine, College of Basic Medical Science, China Medical University, Shenyang 110122, China; 2021120052@cmu.edu.cn (Y.Z.);

**Keywords:** ferulic acid, pharmacological effects, anti-inflammation, toxicology, pulmonary diseases

## Abstract

Ferulic acid (FA), a prevalent dietary phytochemical, has many pharmacological effects, including anti-oxidation and anti-inflammation effects, and has been widely used in the pharmaceutical, food, and cosmetics industries. Many studies have shown that FA can significantly downregulate the expression of reactive oxygen species and activate nuclear factor erythroid-2-related factor-2/heme oxygenase-1 signaling, exerting anti-oxidative effects. The anti-inflammatory effect of FA is mainly related to the p38 mitogen-activated protein kinase and nuclear factor-kappaB signaling pathways. FA has demonstrated potential clinical applications in the treatment of pulmonary diseases. The transforming growth factor-β1/small mothers against decapentaplegic 3 signaling pathway can be blocked by FA, thereby alleviating pulmonary fibrosis. Moreover, in the context of asthma, the T helper cell 1/2 imbalance is restored by FA. Furthermore, FA ameliorates acute lung injury by inhibiting nuclear factor-kappaB and mitogen-activated protein kinase pathways via toll-like receptor 4, consequently decreasing the expression of downstream inflammatory mediators. Additionally, there is a moderate neuraminidase inhibitory activity showing a tendency to reduce the interleukin-8 level in response to influenza virus infections. Although the application of FA has broad prospects, more preclinical mechanism-based research should be carried out to test these applications in clinical settings. This review not only covers the literature on the pharmacological effects and mechanisms of FA, but also discusses the therapeutic role and toxicology of FA in several pulmonary diseases.

## 1. Introduction

**Figure 1:**
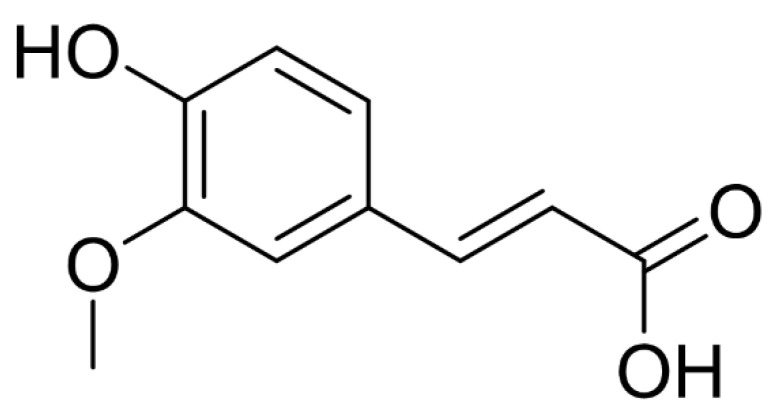
Chemical structure of FA.

Natural products have long been investigated and exploited for the development of new drugs [1]. Ferulic acid (4-hydroxy-3-methoxycinnamic acid, FA) belongs to the phenolic acids commonly found in medical plants (Figure 1). The key physico-chemical properties of FA are shown in Table 1. FA mainly exists in plant cell walls and contributes to the structural integrity and rigidity by being covalently linked to polysaccharides, such as arabinoxylans, which is also a precursor of lignin, a complex polymer that provides mechanical strength and resistance to biodegradation in plant tissues [2,3,4,5]. So far, it has been proven that FA and its derivatives have a variety of pharmacological effects, especially anti-oxidative, anti-inflammatory, anti-allergic, anti-cancer, and anti-fibrotic effects [6,7,8,9,10,11].

FA has been proven to be effective in many disease models through various mechanisms [12,13,14,15,16,17,18]. For example, the nuclear factor-kappaB (NF-κB) and nuclear factor erythroid-2-related factor-2/heme oxygenase-1 (Nrf2/HO-1) signaling pathways are regulated by FA to resist oxidative damage and restrain inflammatory responses, thereby exerting cardioprotective effects [19,20]. Chronic pulmonary diseases are the leading causes of morbidity and mortality worldwide, and the treatment has received increasing attention in recent years. Accumulating evidence suggests that FA improves lung function and survival in pulmonary diseases, including idiopathic pulmonary fibrosis (IPF), acute lung injury/acute respiratory distress syndrome (ALI/ARDS), lung cancer, etc. However, low bioavailability and the limited number of clinical studies have restricted the use of FA in medicine. This review summarizes the main pharmacological effects and mechanisms of FA and discusses its potential role in the treatment of pulmonary diseases.

## 2. Pharmacological Effects

Many researchers have found that FA possesses distinct pharmacological effects, such as anti-oxidative, anti-inflammatory, anti-fibrotic, and anti-cancer effects, as well as complex mechanisms both in vitro and in vivo.

### 2.1. Anti-Oxidative Effects

FA is considered to be a superior antioxidant, which easily forms resonance-stabilized phenoxy radicals due to its phenolic nucleus and an extended side chain conjugation, thus acting as a free radical scavenger [21]. FA can attenuate oxidative stress damage, and the effect is much stronger than vanillic, coumaric, and cinnamic acid [22]. Studies have shown that FA can inhibit the production of reactive oxygen species (ROS), scavenge oxidative free radicals, and participate in various signaling pathways to exert its anti-oxidative effects.

#### 2.1.1. ROS

Oxidative stress arises from impaired endogenous antioxidative defense and/or an overwhelming presence of ROS, which is an important molecular mechanism in various organ pathologies [23,24]. FA treatment can inhibit the production and activity of ROS inducer markers, such as advanced glycation end products and xanthine oxidase [25]. The content of advanced glycation end products and xanthine oxidase activity are evaluated by measuring the immunoblotting data and enzymatic oxidation of xanthine, respectively. In addition, FA can significantly downregulate ROS levels with an ROS-sensitive probe, inhibiting pathological angiogenesis and reducing cell damage [26,27]. In lung cancer, pretreatment with FA initially decreases ROS levels and reduces oxidative damage [28].

#### 2.1.2. Free Radical Scavenging

Previous studies have reported that FA is an antioxidant that neutralizes free radicals such as superoxide, nitric oxide and hydroxyl radicals that may cause oxidative damage to cell membranes and DNA [22]. Due to its structural properties, FA is a direct scavenger of free radicals such as hydroxyl radicals, superoxide, hydrogen peroxide, and nitrogen dioxide radicals [29]. FA has been tested for the radical scavenging property using the 2,2-diphenyl 1-picryl hydrazyl scavenging assay [30]. In a concentration-dependent manner, FA is able to significantly scavenge 2,2-diphenyl 1-picryl hydrazyl free radical with a half-maximal inhibitory concentration (IC_50_) of approximately 33 μM [25]. The generation and detection of hydroxyl radicals are carried out according to a Fenton reaction [31]. Nitric oxide radical scavenging assay has been performed as described by Sumanont et al. [32], with minor modifications. Park and co-worker originally described the procedure used to assess superoxide anion radical production, which was later modified [33]. Catalase activity has been determined essentially using the method described by Aebi [34,35]. Among several tested polyphenols, including ellagic acid, tannic acid, caffeic acid, and FA, the latter is able to suppress the formation of superoxide anion radicals induced by tumor promoters to the greatest extent [36].

The anti-oxidative effect of FA is closely related to the 3-methoxy and 4-hydroxyl groups on the benzene ring, which can stabilize the resulting phenoxy radical intermediates and even terminate the radical chain reaction. The carboxylic acid group with adjacent immature carbon-carbon double bonds can further promote the resonance stabilization of the phenoxy radical intermediates or provide additional attack sites for the radicals [37]. Through this scavenging effect, FA significantly attenuates peroxyl radical-induced cell death and reduces both hydroxyl radical-induced proteins and lipid oxidative damage in hippocampal synaptosomes in vitro [38]. FA efficiently suppresses lipid peroxidation triggered by peroxyl radicals. Additionally, the activity and function of enzymes responsible for scavenging free radicals, such as cardiac superoxide dismutase, glutathione peroxidase, and catalase, are increased by FA [35].

#### 2.1.3. Nrf2/HO-1

**Figure 2:**
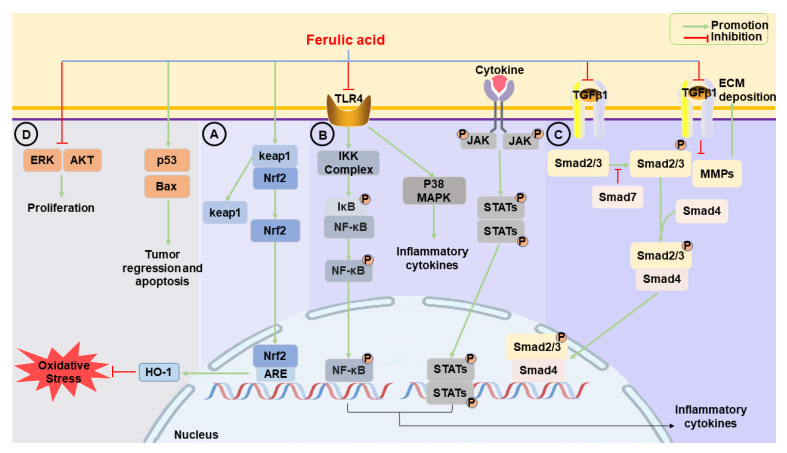
A schematic drawing presenting signaling pathways affected by ferulic acid. (**A**) Under normal conditions, Nrf2 is sequestered in cytoplasm by keap1. Ferulic acid (FA) induces the translocation of Nrf2 from the cytoplasm to the nucleus and promotes the expression of Nrf2, which activates antioxidant response element (ARE) and increases transcription of Nrf2-regulated genes, such as HO-1. FA activates the Nrf2/HO-1 signaling, exerting an anti-oxidative effect. (**B**) The process of activating p38 MAPK and NF-κB signal cascades through TLR4 signaling, leading to the expression of inflammatory cytokines. NF-κB signaling requires IKK subunits, which regulate pathway activation through IκB phosphorylation. The JAK/STAT signaling pathway is activated by cytokines and STATs are dephosphorylated in the nucleus, leading to the activation of downstream inflammatory cytokines. FA acts as an anti-inflammatory agent by inhibiting the p38 MAPK, NF-κB, and JAK/STAT pathways. (**C**) FA can block the activation of TGF-β1/Smads signaling and reverse the nuclear translocation of Smads to resist fibrosis. Furthermore, FA has the ability to alleviate ECM by regulating MMPs. (**D**) FA promotes tumor regression and cell apoptosis by increasing the expression of p53 and Bax, while inhibiting proliferation by decreasing the expression of ERK and AKT. AKT: protein kinase B; Bax: Bcl2-associated X protein; ECM: extracellular matrix; ERK: extracellular signal-regulated kinase; HO-1: heme oxygenase-1; IκB: inhibitor of NF-κB; IKK: IkappaB kinase; JAK: Janus kinase; Keap1: Kelch-like-ECH-associated protein 1; MAPK: mitogen-activated protein kinase; MMPs: matrix metalloproteinases; NF-κB: nuclear factor-kappaB; Nrf2: nuclear factor erythroid-2-related factor-2; Smads: small mothers against decapentaplegics; STAT: signal transducer and activator of transcription; TGF-β1: transforming growth factor-β1; TLR4: toll-like receptor 4.

Nrf2 is one of the main coordinating factors of the oxidative stress response and plays a protective role in many different organs, including the lungs [39], kidneys [40], liver [41], and colon [42]. Nrf2 knockout mice have been reported to be hypersensitive to ALI; their lungs become severely edematous, and microscopy reveals a loss of alveolar structure with pulmonary hemorrhage and infiltration [43]. Under stressed conditions, exposure to toxicants, ROS, genetic mutations, oncogenic signals, or autophagic disruption results in a temporary or constitutive increase in cellular Nrf2, which disrupts the Kelch-like-ECH-associated protein 1 (keap1)-Nrf2 complex, causing Nrf2 activation. Then, Nrf2 dissociates from Keap1 and translocates to the cell nucleus, where it regulates the transcription of target genes, which encode proteins involved in antioxidants, detoxification, anti-inflammation, and metabolism [44,45,46]. FA induces the translocation of Nrf2 from the cytoplasm to nucleus and promotes the expression of Nrf2 as well as downstream antioxidative proteins such as HO-1, playing a cytoprotective role (Figure 2A) [47,48].

FA remarkably prevents nephrotoxicity in rats through activating Nrf2/HO-1 signaling, exerting an anti-oxidative effect [49]. It has been confirmed that FA reverses methotrexate-induced reduction of Nrf2 and HO-1 mRNA in rats with liver injury [50]. Moreover, FA improves alveolar epithelial barrier dysfunction to ameliorate ALI via Nrf2/HO-1 signaling [51]. Similarly, the activation of Nrf2/HO-1 by FA can also have therapeutic potential in alleviating ionizing radiation-induced cataracts [52].

### 2.2. Anti-Inflammatory Effects

Inflammation is the defensive response of the human body when exposed to external stimuli; however, excessive inflammation can result in a variety of diseases [53]. Lipopolysaccharide (LPS) is commonly used in experiments to construct inflammatory pathological models and can be recognized by toll-like receptor 4 (TLR4), which is expressed on the cell surface [54]. The bond of LPS and TLR4 triggers signal transduction cascades in cells, resulting in the activation of NF-κB and mitogen-activated protein kinases (MAPKs), thereby stimulating secretions of inflammatory mediators such as interleukin (IL)-1β, IL-6, and tumor necrosis factor-α (TNF-α) (Figure 2B) [55,56]. Meanwhile, pro-inflammatory cytokines, such as IL-6, IL-11, and IL-13, activate the Janus kinase/signal transducer and activator of transcription (JAK/STAT) molecular pathway to induce inflammation and regulate the immune response [57,58]. One of the most extensively researched inflammasomes, NOD-like receptor-family pyrin domain-containing 3 (NLRP3), can be regulated by NF-κB-induced transcription [59]. FA has shown anti-inflammatory activity through inhibiting these signaling pathways in vitro and in vivo, highlighting its potential as an anti-inflammatory drug.

#### 2.2.1. p38 MAPK

MAPKs are major signal molecules in transduction, catalyzing the phosphorylation of appropriate protein substrates on serine or threonine residues, and playing an important role in the development and progression of inflammation [60,61]. As a member of the MAPKs family, activation of p38 MAPK plays a significant role in the production of pro-inflammatory cytokines such as IL-1β, IL-6, and TNF-α [62,63], and induction of enzymes like cyclooxygenase-2 to regulate connective tissue remodeling, as well as adherent proteins and other inflammatory-related molecules [64,65]. Inflammatory stimuli such as LPS, TNF, platelet activator, and IL can induce p38 activation in endogenous immune cells, such as monocytes, endothelial cells, and neutrophils [66]. The phosphorylation of p38 followed by LPS stimulation is restrained by FA, suggesting that FA may exert anti-inflammatory effects via the inhibition of the p38 MAPK pathway [56,67].

The gene expression of indoleamine 2,3-dioxygenase (IDO) is determined by p38 MAPK, and the activity is induced by cellular immune activation associated with inflammatory diseases [68]. LPS induces the expression of IDO, and FA can pass through the blood-brain barrier to reduce IDO by suppressing the phosphorylation of p38 MAPK, which may provide new ideas for the prevention and treatment of diseases [69]. Moreover, in testicular toxicity induced by cisplatin, a chemotherapeutic drug, pretreatment with FA significantly degrades the expression of p38 MAPK in rats, markedly alleviating cisplatin-induced testicular damage [70].

#### 2.2.2. NF-κB

The NF-κB family of transcription factors contains five members: NF-κB1 (p105/p50), NF-κB2 (p100/p52), RelA (p65), RelB, and c-Rel, among which p65 is the most extensively studied subunit that contains transcriptional activation domains [71,72]. As the major receptor for LPS, TLR4 exists in a complex with co-receptor myeloid differentiation protein-2 [73,74]. Upon binding to LPS, the TLR4-myeloid differentiation protein-2 complex dimerizes, which leads to the activation of downstream mediators, including NF-κB [75]. Stimulation triggers degradation of the inhibitor of NF-κB (IκB) protein and release of NF-κB homo- or heterodimers, which subsequently translocate to the nucleus, then bind to specific DNA sequences and promote the transcription of pro-inflammatory genes [76]. Treatment with FA significantly inhibits the expression of LPS-induced TLR4, degradation of IκB, and phosphorylation of p65. Docking results have shown that FA targets the key binding site of TLR4 and disrupts the formation of the TLR4-myeloid differentiation factor 2 complex, which provides a new strategy for the treatment of inflammation [77]. In addition, researchers have found that FA markedly prevents IκB phosphorylation and subsequent nuclear translocation of NF-κB [78,79]. FA treatment has the same effect as TLR4 inhibitor (TSK242) and NF-κB inhibitor (SP600125), giving it the potential to act as an effective inhibitor of inflammation [77].

#### 2.2.3. JAK/STAT

Various cytokines and growth factors can bind to their respective receptors on the cell surface and lead to the phosphorylation and activation of JAK kinases and STATs sequentially [80]. The latter translocate to the nucleus and regulate the expression of genes involved in cell proliferation, differentiation, survival, and inflammation (Figure 2B) [81]. Accordingly, the JAK/STAT signaling system may be a useful indicator of a strong immune response, inhibiting of which could help to reduce hyperinflammatory conditions [82]. FA has been found to have promising JAK2 inhibition through molecular docking with a score of −6.7, which is comparable to that of ruxolitinib, a standard JAK2 inhibitor [83].

#### 2.2.4. NLRP3

The NLRP3 inflammasome is a cytosolic protein complex that senses cellular stress or damage and initiates inflammatory responses, which has been implicated in various pulmonary diseases, such as asthma and chronic obstructive pulmonary disease (COPD) [84,85]. Therefore, inhibiting the activation of NLRP3 inflammasome is a potential strategy for preventing or treating inflammation-related diseases [86]. Studies have shown that FA can exhibit anti-inflammatory effects by blocking the activation of NLRP3 and reducing the secretion of TNF-α, IL-1β, and IL-6 [87,88].

### 2.3. Anti-Fibrotic Effects

Fibrosis is a repair or reactive process characterized primarily by the formation of fibrous connective tissue, resulting in progressive structural remodeling of almost all tissues and organs [89]. For example, the pathogenesis of pulmonary fibrosis (PF) arises from repeated damage to the alveolar epithelium or endothelium, triggering the immune system to recover the tissue structure of the injured tissue. Inflammatory mediators, such as transforming growth factor-β (TGF-β), are able to activate angiogenesis and myofibroblasts, which promote the generation of extracellular matrix (ECM) constituents [90,91]. Excessive accumulation of ECM and promotion of fibrosis through endogenous and exogenous stimuli that induce elevation of TGF-β1 exacerbate the imbalance between matrix metalloproteinases (MMPs) and tissue inhibitors of metalloproteinases (TIMPs) [92,93]. When ECM is overproduced and deposited in organ tissues, substantial scar formation and destruction of normal organ architecture occurs [94]. Almost all lung diseases end in fibrosis, but an effective cure for fibrosis has yet to be found. In some early reports, FA was found to be helpful in suppressing fibrosis [95,96].

#### 2.3.1. TGF-β/Small Mothers against Decapentaplegic

TGF-β is a multifunctional regulatory cytokine known to regulate various cellular processes, such as proliferation, differentiation, apoptosis, adhesion, and the pathogenesis of fibrosis, in which TGF-β1 is a key mediator of fibrosis development, exerting biological effects through activation of downstream mediators [97]. After the downstream small mothers against decapentaplegic (Smad)2 and Smad3 are activated, they form a complex with Smad4 that translocates to the nucleus, binds consensus sequences, and regulates gene transcription, which induces ECM deposition to promote fibrosis (Figure 2C) [98,99,100].

FA can block the activation of the Smad2/3 signal, reverse Smad4 nuclear translocation, inhibit the epithelial–mesenchymal transition process, which is driven by TGF-β1, and resist the occurrence of fibrosis [101,102]. In addition, it has been found that FA can decrease Smad3 and Smad4 by inhibiting the expression of TGF-β and its receptor and can cooperate with astragaloside IV to alleviate fibrosis in rats [103]. Based on an in vitro study, TGF-β signal transduction can be blocked by FA, which significantly reduces Smad signal transduction to inhibit the activation of hepatic stellate cells [95].

#### 2.3.2. MMPs/TIMPs

The MMPs/TIMPs system has been reported to be regulated by FA [104]. MMP2 and MMP9 have three fibronectin type II structural domains repeatedly inserted into the catalytic structural domain, which are closely associated with the development of fibrosis [105]. MMP1 is involved in the diminution of normal and hypertrophic scars [106]. TIMPs play essential roles in the activation or elimination of MMPs from the extracellular environment, which determines the effects of ECM on cytokines, chemokines, cell adhesion molecules, and growth factors [107]. TIMP1 is secreted by most cells and inhibits all types of MMPs, among which TIMP1 binds particularly strongly to MMP9 [108]. FA has displayed reductions in MMP2 and MMP9, and an increase in TIMP1 expression [109]. One study has shown that FA may lead to a significant reduction in MMP2 and MMP9 levels via the proteasome pathway [110].

### 2.4. Anti-Cancer Effects

Cancer is a serious disease that causes deaths all over the world, and the incidence and mortality rates are increasing rapidly [111]. Evading apoptosis has been established as one of the key characteristic features of cancer cells [112]. Cancer proliferation can be inhibited by FA in a variety of ways, including by altering the cancer cell cycle, inducing apoptosis, and regulating protein production [113].

FA prevents migration in breast cancer cells and so suppresses breast cancer cell proliferation and induces apoptosis [114]. In addition, many studies have shown that FA has anti-cancer effects against cervical cancer [115], colon cancer [116], liver cancer [28], and lung cancer [117]. ElKhazendar et al., have studied the therapeutic effect of FA on liver cancer and found that FA (100 and 200 μg/mL) has cytotoxic effects on HepG2 cells with IC_50_ values of 150.7, 81.38, and 210.4 μg/mL at 24, 48, and 72 h, respectively [118]. They have reported similar findings after analyzing the anti-cancer potential of FA (100 and 200 μg/mL) on MCF-7 breast cancer cells in vitro and found IC_50_ values of cell proliferation for MCF-7 cells of 143.8, 75.4, and 85.6 μg/mL at 24, 48, and 72 h, respectively [118]. It is reported that FA can take on an anti-cancer role by mediating different targets.

#### 2.4.1. p53

Genes involved in cancer development can be divided into oncogenes and tumor suppressor genes [119]. As one of the most important tumor suppressor genes involved in cell cycle control and induction of apoptosis following DNA damage and oncogene activation, p53 is activated, stabilized, and accumulated by post-translational modifications in the cell, which reduces the risk of tumorigenesis [120]. Many different cell biological responses are induced by p53, such as G1 arrest, senescence, and apoptosis [121]. p53 has been shown to promote apoptosis, whereas overexpression of cyclin D1 leads to a shorter duration of the G1 phase and accelerates cancer progression [122]. The protein level of cyclin D1 is decreased and p53 is upregulated after FA treatment, indicating that FA arrests the G0/G1 phase in human cervical cancer cells [115,123]. Additionally, FA can ameliorate placental apoptosis in a preeclampsia rat model by facilitating B cell lymphoma-2 (an anti-apoptotic protein) expression and decreasing the expression of Bcl2-associated X protein (Bax), which is a pro-apoptotic effector (Figure 2D) [124]. FA derivatives also inhibit cell proliferation, and also induce cell cycle changes and apoptosis. Hexyl ferulate acts mainly through a mitochondrial pathway involving p53 and Bax, resulting in increased cell death and restrained development of cancer [125].

#### 2.4.2. Extracellular Signal-Regulated Kinase

Extracellular signal-regulated kinase (ERK) targets different molecules to stimulate cell proliferation and plays a crucial role in regulating physiological processes such as cell growth, proliferation, and apoptosis. The inactivation of ERK can upregulate or downregulate expression of pro-apoptotic proteins and survival proteins, respectively [119,126]. Sustained activation of ERK can promote proliferation and migration of tumor cells. FA is capable of inhibiting the overexpression of p-ERK1/2 and ERK1/2 proteins, thus exerting a proliferation-inhibiting effect (Figure 2D) [127].

FA inactivates ERK1/2 and c-Jun N-terminal kinase (JNK), so as to inhibit angiotensin II-induced proliferation of vascular smooth muscle cells, thereby reducing the expression of cell cyclin D1 and regulating the process of cells from the G1 to S phase [128,129]. Furthermore, a FA derivative activates the JNK signaling pathway, while inhibiting the ERK signaling pathway, and induces apoptosis in lung cancer cells [130].

#### 2.4.3. Protein Kinase B

Protein kinase B (AKT) is a serine threonine kinase that mediates various biological functions such as cell proliferation, survival, glucose metabolism, protein synthesis, genome stabilization, and inhibition of apoptosis in response to different growth factors and extracellular stimuli. Many studies have shown that one of the corporate molecular features of human malignancies is excessive activation of AKT, leading to tumor aggressiveness and drug resistance [131,132]. Treatment with FA inhibits the proliferation of osteosarcoma cells (IC_50_ = 59.88 μM) and promotes the apoptosis by downregulating the expression and activation of AKT (Figure 2D) [133,134]. Additionally, inhibitory effects of FA on angiogenesis and cell proliferation have been demonstrated in vitro and in vivo, with significant inhibition of FGFR1-mediated AKT phosphorylation by FA [135,136]. FGFR1 kinase activity has been directly inhibited by FA in a dose-dependent manner with an IC_50_ of approximately 3.78 μM.

#### 2.4.4. Programmed Cell Death

Programmed cell death (PCD) is a process that regulates the elimination of unwanted or damaged cells in a controlled manner, which can be classified into apoptosis, necrosis, autophagy, and ferroptosis, etc. [137,138]. PCD plays an important role in maintaining tissue homeostasis and preventing tumorigenesis, but nonetheless results in various pathological conditions [139,140]. One of the PCD types associated with pulmonary diseases is ferroptosis, which is caused by an increase in iron-dependent ROS due to intracellular iron overload, leading to lipid peroxidation and cell membrane damage [141]. Ferroptosis has been shown to be involved in the pathogenesis and progression of ALI/ARDS, lung cancer, PF, and asthma [142,143,144,145]. FA has been shown to inhibit ferroptosis by modulating several key factors involved in this process, such as glutathione peroxidase 4, Nrf2, and adenosine monophosphate-activated protein kinase [146]. Another type of PCD related to pulmonary diseases is apoptosis mediated by programmed cell death 4 (PDCD4), which encodes a tumor suppressor protein that inhibits translation initiation and promotes apoptosis [147,148]. PDCD4 is frequently downregulated in lung cancer, and the expression is associated with tumor progression and prognosis [149]. FA may inhibit the degradation of PDCD4 protein by preventing its phosphorylation through the mechanistic targeting of rapamycin/ribosomal protein S6 kinase 1 [150,151].

## 3. Therapeutic Effects on Pulmonary Diseases

**Figure 3:**
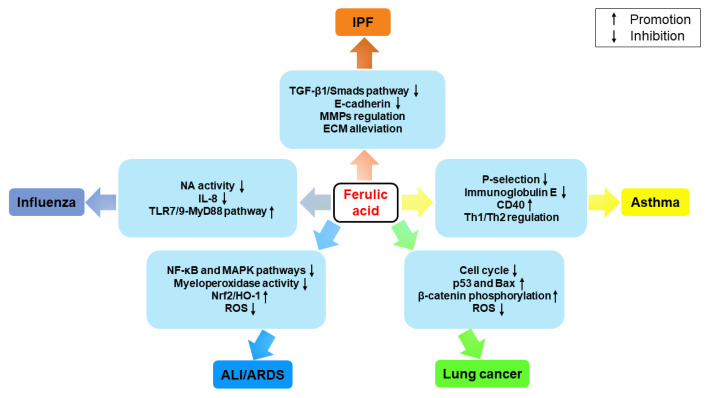
Pharmacological effects associated with ferulic acid in different pulmonary diseases. ALI/ARDS: acute lung injury/acute respiratory distress syndrome; Bax: Bcl2-associated X protein; ECM: extracellular matrix; HO-1: heme oxygenase-1; IL-8: interleukin-8; IPF: idiopathic pulmonary fibrosis; MAPK: mitogen-activated protein kinase; MMPs: matrix metalloproteinases; MyD88: myeloid differentiation primary response 88; NA: neuraminidase; NF-κB: nuclear factor-kappaB; Nrf2: nuclear factor erythroid-2-related factor-2; ROS: reactive oxygen species; Smads: small mothers against decapentaplegics; TGF-β1: transforming growth factor-β1; TLR7/9: toll-like receptor 7/9.

Pulmonary diseases, including IPF, asthma, lung cancer, ALI/ARDS, influenza, etc., rank amongst the most common causes of death globally [152]. Both the incidence and mortality rate of lung cancer are at the top of the list among all malignant tumors [153]. The prevalence of IPF and asthma is increasing, constituting a significant threat to public health. Available therapeutic approaches for pulmonary diseases focus on relieving symptom severity and enhancing quality of life; existing treatments are unable to achieve complete recovery of lung function [154]. As a possible treatment for end-stage pulmonary diseases, lung transplantation has a high mortality rate; thus, novel therapies are urgently needed [155,156,157]. In recent years, there has been an increasing number of studies on the therapeutic effects of FA in pulmonary diseases (Figure 3).

### 3.1. IPF

As a fatal and incurable lung disease with increasing incidence, IPF can be restricted by FA, which reduces the migration of inflammatory cells, deposition of excessive ECM components, and secretion of pro-inflammatory cytokines such as IL-1β, IL-6, and TNF-α [96,101,158].

**Figure 4:**
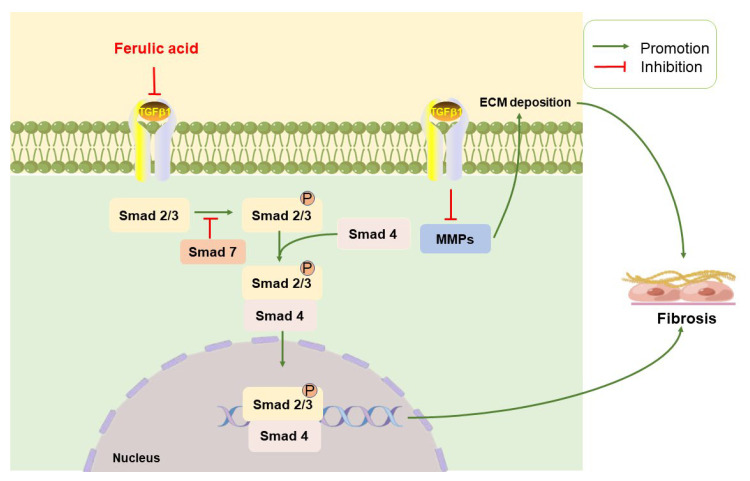
Proposed signaling pathway for the effect of ferulic acid against pulmonary fibrosis. In pulmonary fibrosis, transforming growth factor-β (TGF-β) triggers small mothers against decapentaplegic (Smad) 2/3, of which the phosphorylated form compounds with Smad4, and then enters into the nucleus to regulate gene transcription by binding to cofactors or DNA sequences. In addition, TGF-β inhibits the deposition of ECM by suppressing MMPs. Ferulic acid inhibits the TGF-β signaling pathway to alleviate pulmonary fibrosis. ECM: excessive extracellular matrix; MMPs: matrix metalloproteinases.

Several fibrotic proteins are known to exacerbate PF, among which TGF-β enhances ECM deposition, promotes epithelial–mesenchymal transition, and induces fibroblast differentiation [159,160,161]. FA can inhibit the TGF-β1/Smad3 signaling pathway by downregulating the phosphorylation of Smad2/Smad3 and can block the TGF-β mediated downstream regulation of epithelial marker E-cadherin, so as to alleviate PF [96,162]. Of note, FA alleviates TGF-β induced ECM production through the Smad3-dependent/non-dependent pathway, i.e., MMPs (Figure 4) [163,164]. As a component of the Yangfei Huoxue Decoction, FA restrains vascularized vascular endothelial growth factor and IL-1β expression, indicating a possible protective effect in PF treatment [165,166].

### 3.2. Asthma

Asthma is a chronic inflammatory disorder of the respiratory tract, characterized by mucus hypersecretion, airflow limitation, bronchial hyperresponsiveness, and airway inflammation [167,168]. FA can relieve several allergic complications by exerting immunomodulatory effects, such as cutaneous anaphylaxis in an allergic mouse model, suggesting that FA is a promising candidate for the effective control of asthma [8,169]. It has also been demonstrated that FA decreases the expression of P-selectin on the platelet surface and reduces airway inflammation, which can inhibit endothelial cell adhesion and improve lung function in asthma [166,170]. FA can reduce immunoglobulin E and activate dendritic cells via enhancing the expression of CD40, then restoring the T helper cell (Th)1/Th2 imbalance [8,171]. Recently, in order to better exert the anti-asthma effect, FA is packaged into chitosan-based nanocarriers to ensure drug delivery to epithelial cells [172].

### 3.3. Lung Cancer

Globally, lung cancer is the most common cancer and the leading cause of cancer deaths, of which approximately 80% are non-small cell lung cancer (NSCLC) [111,173]. FA has been used to enhance the sensitivity of cancer cells to radiation, with low systemic toxicity [174,175]. FA treatment given along with radiation is able to arrest the cell cycle, increase the expression of the pro-apoptotic proteins p53 and Bax, and inhibit the anti-apoptotic capacity of A549 and NCI-H460 cells [28,176]. FA derivatives can limit the proliferation and metastasis of lung cancer by reducing the phosphorylated expression of ERK, AKT, and MAPK kinases, which have been shown to be involved in cell invasion and are associated with reduced survival rates in a variety of human malignancies [130]. Trans-FA inhibits the proliferation of H1299 lung cancer cells and induces a moderate increase in the apoptotic population by promoting phosphorylation of β-catenin at residues Thr41 and Ser45 and causing proteasomal degradation [117,177]. Intriguingly, FA is capable of inhibiting the proliferation and migration of lung cancer cells by eliminating intracellular ROS production in tumor cells and of slowing tumor progression by suppressing the adhesion and migration of A549 lung cancer cells [117,178].

### 3.4. ALI/ARDS

**Figure 5:**
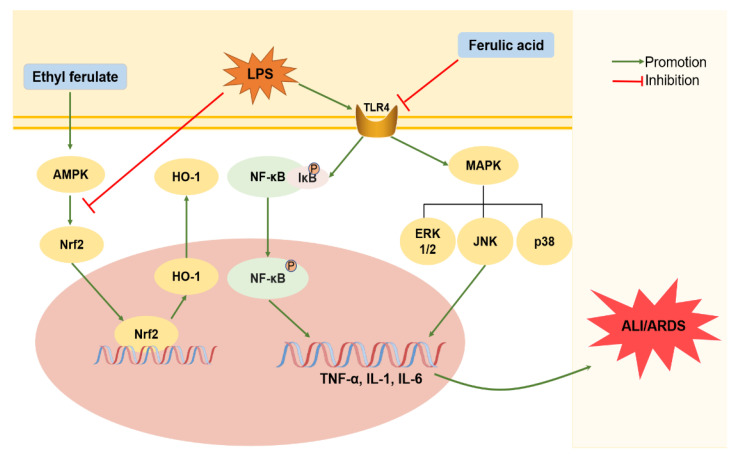
Insights into the multi-target actions of ferulic acid in acute lung injury/acute respiratory distress syndrome. Ferulic acid (FA) can directly target TLR4 and inhibit the TLR4/NF-κB pathway, decreasing the expression of phospho-NF-κB and downstream inflammatory mediators. Moreover, FA inhibits the expression of MAPK signaling pathway-related proteins. Additionally, ethyl ferulate improves acute lung injury/acute respiratory distress syndrome (ALI/ARDS) in an AMPK/Nrf2-dependent manner. AMPK: adenosine monophosphate-activated protein kinase; HO-1: heme oxygenase-1; MAPK: mitogen-activated protein kinase; Nrf2: nuclear factor erythroid-2-related factor-2; TLR4: toll-like receptor4.

ALI and ARDS are successive lung changes arising from multifarious lung injuries with significant morbidity and mortality, which are characterized by bursts of inflammation and damaged alveolar-capillary structures [179,180]. FA can ameliorate ALI by inhibiting the NF-κB and MAPK pathways via TLR4 and consequently decreasing the expression of downstream inflammatory mediators, including TNF-α, IL-1β, IL-6, and IL-8 (Figure 5) [77,181,182]. FA also downregulates the activity of myeloperoxidase, an indicator of neutrophil infiltration [181]. As a derivative of FA, ethyl ferulate inhibits the production of inflammatory mediators in LPS-stimulated macrophages, which also block the translocation of NF-κB p65 to the nucleus and significantly reduce intracellular ROS levels [183]. The activity of superoxidase dismutase, which is an anti-oxidative enzyme that scavenges superoxide radicals, is significantly enhanced in the treatment of sodium ferulate [184]. Furthermore, FA has been found to prevent ARDS by inhibiting the expression of MAPK signaling pathway-related proteins, including p-p38, p-ERK1/2, and p-JNK (Figure 5) [185]. Growing evidence indicates that lung inflammation and injury are regulated by adenosine monophosphate-activated protein kinase [186], which can be activated by ethyl ferulate in a Nrf2/HO-1 dependent manner [183,187].

### 3.5. Influenza

Influenza is an acute viral respiratory infection with a high morbidity rate [188]. Current strategies for treating influenza focus on inhibiting the function of neuraminidase (NA), one of the surface proteins of the virion, which supports the release of progeny virions from the host cells and their movement to target cells [189]. FA has moderate NA inhibitory activity and shows a tendency to reduce the downstream IL-8 level in response to influenza virus infections [190]. The ring structure of FA is similar to that of the NA inhibitor oseltamivir, such as in the C1 and C5 positions [190,191]. In contrast, at the C3 position, there is no semblable amino group in FA. Therefore, slight structural modifications of FA, such as the introduction of an amino substituent into the guanidine group, could improve FA activity, which would eventually increase NA inhibition in vitro. A virus inhibition experiment shows that the FA derivative MY15 has good activity, with a median effective concentration of about 0.95 μM [190]. Beyond that, the protective immune response to influenza is controlled by the TLR7/TLR9-myeloid differentiation primary response 88 pathway, which is enhanced by sodium ferulate in mice [192].

### 3.6. Other Pulmonary Diseases

COPD is a progressive lung disorder characterized by oxidative stress, inflammation, endothelial dysfunction, fibrosis, and apoptosis [193,194]. As one of the bioactive components of Bu-Zhong-Yi-Qi-Tang, FA has been shown to reduce the levels of TNF-α and IL-6, as well as prevent neutrophil and macrophage infiltration by downregulating cell-adhesion molecules, such as P-selectin, which may help to relieve the symptoms of COPD [195].

Pneumoconiosis is a group of serious occupational diseases which are associated with the inhalation of mine dust and the corresponding reaction of the lung tissue [196,197]. Sodium ferulate can inhibit the activation of the TGF-β1/neutrophilic alkaline phosphatase 3/α-smooth muscle actin pathway, which provides a potential therapeutic strategy for silicosis-associated PF [197].

As one of the major components of Rhodiola algida, which prevents high latitude sickness clinically, FA is effective in hypoxia-induced pulmonary arterial hypertension animals [198]. Similarly, sodium ferulate has been used clinically in the treatment of pulmonary hypertension with satisfactory results [199].

The severe acute respiratory syndrome coronavirus-2 (SARS-CoV-2) caused the outbreak of coronavirus disease 2019, against which the therapeutic target is the SARS-CoV-2 main protease [200]. Some FA derivatives, such as FA rutinoside and raffinose ferulate, show comparable or better binding affinities for the main protease of SARS-CoV-2 as confirmed inhibitors, and might have antiviral potential against coronavirus disease 2019 [201].

## 4. Toxicological Effects

At a dose of 300 μg/mL, FA has no effect on the cell count and viability of platelets, leukocytes, and erythrocytes. There is hardly any toxicity of FA to NIH-3T3 and 3T3-L1 cells at the concentration of 500 μg/mL [202]. Nevertheless, studies have demonstrated the renal-damaging effect of FA when used for the 28-week cure of chronic kidney diseases [203]. Intriguingly, the toxicity of FA is seasonally dependent, being more toxic in May, June, and September, which may be related to abiotic factors, including carbon dioxide, temperature, and pH [204].

## 5. Conclusions and Future Directions

FA and its derivatives are currently being used with breakthrough results in various fields. Modern pharmacological studies have proven that FA has a variety of effects, such as anti-oxidative, anti-inflammatory, anti-fibrotic and anti-cancer effects. Firstly, as a free radical scavenger, FA significantly downregulates ROS expression and activates Nrf2/HO-1 signaling, exerting anti-oxidative effects. Secondly, FA acts as an anti-inflammatory agent by inhibiting the p38 MAPK, NF-κB, and JAK/STAT pathways. The TGF-β/Smad signaling pathway can be blocked by FA, which plays an anti-fibrotic role. The MMPs/TIMPs system can be regulated by FA to inhibit the expression of MMP2 and MMP9, thereby enhancing the anti-fibrotic effect. Ultimately, the anti-cancer effect of FA is closely connected with p53 upregulation, Bax downregulation, and inactivation of ERK and AKT.

In addition to providing a summary of the pharmacological mechanisms of FA, we also consider the therapeutic advances in pulmonary diseases. FA has shown significant promise in the field of lung disease treatment. Initially, by blocking the TGF-β1/Smad3 signaling pathway and inhibiting MMPs expression, FA plays a role in improving IPF. Furthermore, FA reduces the expression of P-selectin and restores the Th1/Th2 imbalance, exerting an anti-asthma effect. With regards to lung cancer, the expression of p53 and ROS production are regulated by FA. Thirdly, FA has prevented ARDS through inhibiting the expression of MAPK signaling pathway-related proteins, including p-p38, p-ERK1/2, and p-JNK. Additionally, FA has a moderate NA inhibitory activity, which shows a tendency to reduce downstream IL-8 levels in response to influenza virus infections.

One of the main limitations of the clinical application of FA to date has been low bioavailability, and most of the technological strategies used to improve the oral bioavailability of FA are based on lipid delivery systems [205]. Both nanostructured lipid carriers and solid lipid nanoparticles can enhance the oral bioavailability of FA [206]. In addition, a major issue in the drug-discovery process is toxicity. Thus, FA, with its low toxicity properties, is a very valuable natural compound with potential for the treatment of pulmonary diseases.

This review discusses the pharmacological effects, applications in pulmonary diseases, and toxicology of FA to heighten our understanding of existing research. More in-depth research is needed to explore the molecular mechanisms of FA, in order to provide an efficient scientific basis for enlarging the scope of clinical treatment and exploiting the potential of FA in drug applications.

## Figures and Tables

**Table 1:** Physico-chemical properties of FA.

Property	Index
Molecular weight	194.18
Melting point	170.0 ± 2.0 °C
Boiling point	372.3 ± 27.0 °C
Density	1.316 ± 0.06 g/cm^3^
Cis isomer	Yellow oily substance
Trans isomer	Monoclinic crystal
Solubility	Soluble in hot water, ethanol and ethyl acetate, slightly soluble in diethyl ether, and poorly soluble in benzene and petroleum ether

## Data Availability

No new data were created or analyzed in this study. Data sharing is not applicable to this article.
